# Imaging the brain in action: a motorized optical rotary joint for wide field fibroscopy in freely moving animals

**DOI:** 10.1117/1.NPh.10.1.015009

**Published:** 2023-03-24

**Authors:** Timothé Jost-Mousseau, Max Chalabi, Daniel E. Shulz, Isabelle Férézou

**Affiliations:** Université Paris-Saclay, Centre National de la Recherche Scientifique, Institut des Neurosciences Paris-Saclay (NeuroPSI), Saclay, France

**Keywords:** optical rotary joint, mesoscopic functional imaging, cerebral cortex, freely behaving animals, voltage sensitive dye imaging, inertial measurement unit

## Abstract

**Significance:**

The study of neuronal processes governing behavior in awake behaving mice is constantly boosted by the development of technological strategies, such as miniaturized microscopes and closed-loop virtual reality systems. However, the former limits the quality of recorded signals due to constrains in size and weight and the latter suffers from the restriction of the movement repertoire of the animal, therefore, hardly reproducing the complexity of natural multisensory scenes.

**Aim:**

Another strategy that takes advantage of both approaches consists of the use of a fiber-bundle interface to carry optical signals from a moving animal to a conventional imaging system. However, as the bundle is usually fixed below the optics, its torsion resulting from rotations of the animal inevitably constrains the behavior over long recordings. Our aim was to overcome this major limitation of fibroscopic imaging.

**Approach:**

We developed a motorized optical rotary joint controlled by an inertial measurement unit at the animal’s head.

**Results:**

We show its principle of operation, demonstrate its efficacy in a locomotion task, and propose several modes of operation for a wide range of experimental designs.

**Conclusions:**

Combined with an optical rotary joint, fibroscopic approaches represent an outstanding tool to link neuronal activity with behavior in mice at the millisecond timescale.

## 1 Introduction

Unraveling the links between neural activity and behavior is a major challenge to progress in our comprehension of the normal and pathological functioning of the central nervous system.

During the last two decades, the tremendous expansion of the molecular toolbox allowing to probe^1^^–^^5^ and control^6^ neuronal activity with light^7^^,^^8^ has fostered the development and use of “optophysiological” techniques in integrative neurosciences. This has been accompanied by an intensification of the use of the mouse as a model system, as it facilitates the implementation of these molecular tools and offers a rich repertoire of behaviors. Meanwhile, a constant effort has been made to push forward optical methods, in quest for a better resolution in space and time, on ever wider fields of view.

Altogether, these technological means that one could gather under the term “optophysiology” are complementary to electrophysiological approaches and comparatively offer some additional advantages, such as their insensitivity to electromagnetic disturbances, or the ability to reduce tissue invasiveness in the outermost structures of the brain. The major strength of methods based on genetically encoded probes or actuators is to enable the identification of the source of activity in the neural tissue. On the other hand, synthetic voltage-sensitive dyes (VSDs) are to date the most potent means to measure ensemble neuronal activity with high temporal (down to 1 ms) and spatial (<50  μm) resolution over large fields of view.^9^

The development of these optophysiological approaches has rapidly raised the question of coupling optical recording or patterned photo-stimulation with the performance of complex behavioral tasks in mice. Currently, three main experimental strategies have been adopted to deal with this issue.

The first one consists in training mice to be held firmly by their head under sophisticated optical systems. However, this condition restricts the behavioral repertoire under study. Head fixation is indeed a strong constraint when designing protocols to interrogate how central neuronal networks predict and integrate sensory information or generate motor commands on the basis of environmental cues and prior experiences. This difficulty has been partially addressed through the implementation of computer-controlled closed-loop “virtual reality” environments around the head-fixed mouse standing on a spherical^10^^,^^11^ or linear^12^^,^^13^ treadmill. Their locomotion can be monitored in real time to dynamically affect screens,^10^^,^^11^ actuators,^12^^,^^14^ or other means,^15^ simulating an environment that changes accordingly by stimulating different sensory modalities. Note that simpler systems have been implemented, using only the mechanical forces produced by the animal’s motility to update its environment, such as an air-lifted mobile cage^16^ or a treadmill consisting of a long belt containing both visual and tactile cues.^17^ All these approaches allow to use the entire range of cutting-edge optical methods and provide great control over the experimental paradigm by reducing degrees of freedom of the experimental subject and its sensory environment.^18^ However, the richness of behaviors that can be studied, and the fidelity of sensory stimulations compared to the real-world experience of the subject, do not cover the huge parametric space of natural conditions. Furthermore, the physiological bases of some neural functions such as those involving the vestibular system, social interactions, or prey capture^19^ can hardly be studied in this framework.

A second strategy relies on the use of miniaturized optical devices embedded on top of the mouse’s head.^20^^,^^21^ Most of these devices are one-photon optical systems, which require only electrical connections for power supply and data collection. Hence, they permit real mobility of the animal, which can be optimized simply through the use of a slip ring electrical commutator. Such mini-one-photon microscopes enable optical imaging from the cellular scale in the cerebellar cortex and deep subcortical structures,^22^^–^^24^ up to the mesoscopic scale over the whole dorsal part of the cerebral cortex.^25^ They can further incorporate a light source for bulk opsin photo-activation.^26^ Recently, a portable digital micromirror device (DMD) has been implemented on a one-photon mini-microscope to allow patterned opsin photoactivation^27^ in freely moving mice, but this device requires an additional optical fiber for light delivery to the DMD. In parallel with the development of miniaturized one-photon microscopes, efforts have been made to assemble mini-multiphoton microscopes that can be mounted on the head of freely-moving mice. However, in addition to electrical connections, these systems also require the use of an optical fiber to bring the excitation laser beam to the imaged surface,^28^^,^^29^ and, for one of them, an image guide (tapered fiber-bundle) to collect the emitted photons.^29^ These optical cables constitute an extra difficulty in allowing the mice to navigate and rotate freely in their environment. In essence, embedded optical systems often suffer from the miniaturization process in terms of quality of the output signal of the device, while representing a fairly large mass and volume compared to the size of a mouse.

The third approach consists in the use of optic-fiber bundles, made of arrays of individual fibers packed together to transfer a spatially organized optical signal between the animal and an optical system that can remain static. This approach benefits from the advantages of the two previous ones. On one hand, the possibility to use any kind of sophisticated and bulky optical system, and on the other hand, to allow a real mobility of the animal. However, these are gained at the cost of a spatial discretization of the optical signal, which depends on the number of individual fibers present in the bundle. As with miniature microscopes, this is a rapidly evolving field, which has proven its potential for optical imaging of neuronal activity both at the mesoscopic scale in the cerebral cortex,^30^ at the cellular scale in subcortical structures,^31^^,^^32^ and for multisite photometry^33^^,^^34^ in freely moving mice. The fibroscopic approach has also been used successfully to apply patterned photoactivation at the cellular scale in freely moving mice.^35^

In the same way as for the recently developed miniaturized two-photon microscope mentioned above, these methods rely on a bundle of optical fibers, which accumulates torsion as rotations of the animal increase, therefore constraining the behavioral protocols under study. Unlike for electrical signals where rotary joints are very commonly used to address this issue, the rotative unaltered transmission of coherent optical signals still remains a challenge.

To solve this problem, we have developed a robust and relatively inexpensive motorized optical rotary joint that can be either coupled in real time to the rotation of the animal’s head by means of an embedded inertial measurement unit (IMU), or locked at will in front of the image acquisition system. In doing so, it advantageously allows combining optimization of the animal’s mobility with best imaging performances. Here we describe this device and illustrate its use for VSD imaging of cortical activity in the barrel cortex of mice engaged in a whisker-guided locomotion task. Moreover, the optical rotary joint can be used in different operating modes, which should offer optimal use for a wide range of imaging methods and behavioral tasks.

## 2 Methods

### 2.1 Apparatus

**Fig. 1:**
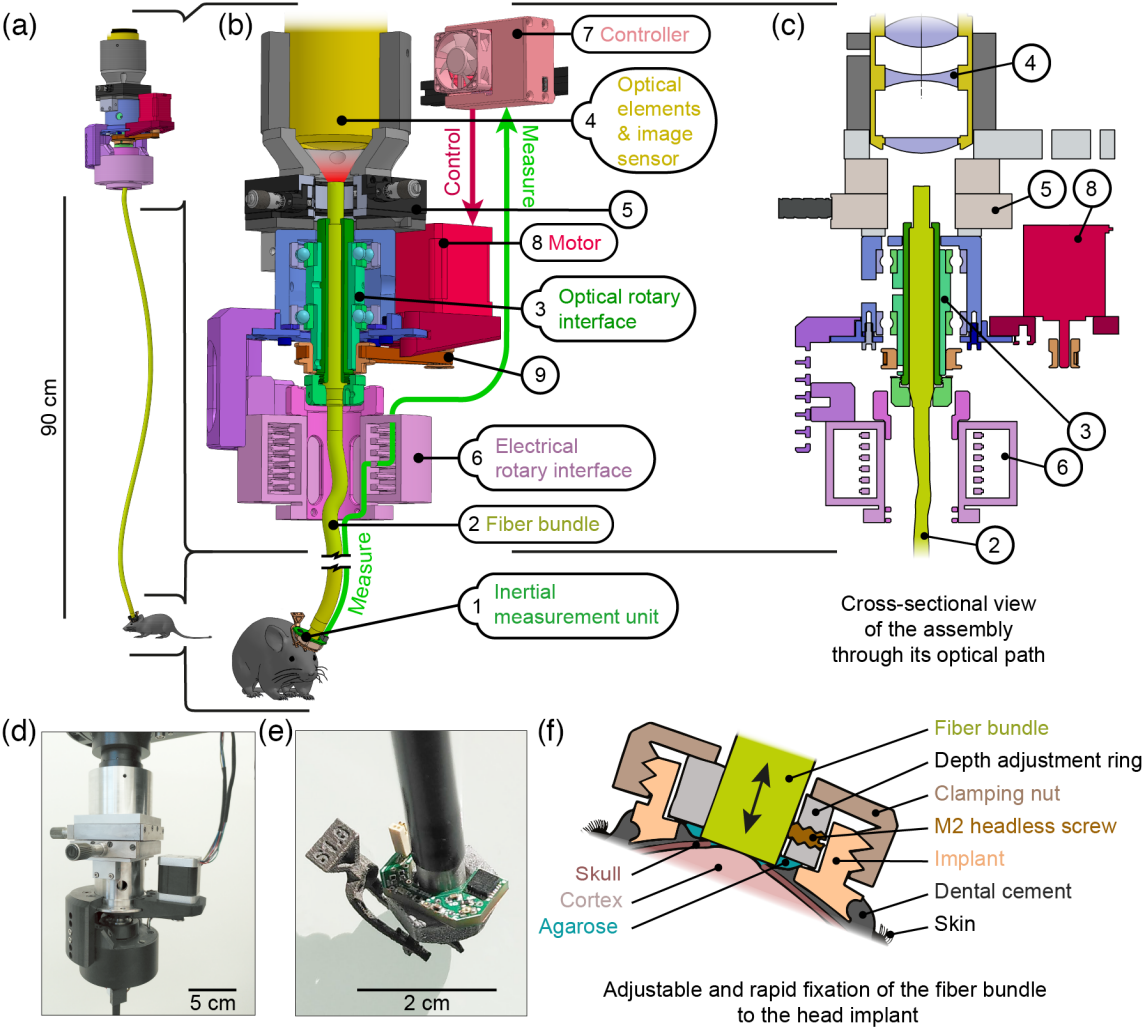
Motorized rotary optical interface coupled to target’s absolute orientation. (**a**) Schematic representation of the device in its real proportions. (**b**) Detailed diagram of the apparatus assembly. 1: IMU, 2: fiber-bundle, 3: optical rotary interface, 4: optical elements and image sensor, 5: 2D translation system for fine adjustment of the fiber-bundle proximal end relative to the sensor, 6: electrical rotary interface, 7: controller, 8: stepper motor, and 9: timing belt transmission. (**c**) Cross-sectional view of the assembly though its optical axis. (**d**) Photo of the rotary joint. (**e**) Photo of the distal end of the fiber-bundle, on which the implant and IMU are attached. (**f**) Cross-sectional view of the skull and cortex of a mouse surmounted by the implant components allowing the fixation of the fiber bundle in close contact with the cortical surface. The clamping nut allows a rapid fixation of the bundle in the desired position which is precisely set by means of the depth adjustment ring.

To enable fibroscopic neuronal imaging in freely behaving mice with minimal movement restrictions, we have implemented an active rotating optical interface, which can follow any kind of user commands, or more specifically match the absolute orientation of the animal computed in real time [Figs. 1(a)–1(d)].

Optical imaging is achieved through a 90-cm-long coherent fiber-bundle interface (Schott AG, wound fiber-bundle, 0.6 numerical aperture) which is fixed to the animal’s head by means of a custom designed head implant [0.8 g, Titanium, 3D printed by Sculpteo, Figs. 1(e) and 1(f)].

Importantly, the head implant design allows the fixation, on the mouse, of a small, 9 degrees-of-freedom, IMU [InvenSense ICM20948, Fig. 1(b1)]. This IMU comprises a magnetometer, which is key to correct for accelerometer and gyroscope error accumulations over time.

The distal end of the fiber-bundle is inserted into the animal’s head-implant at adjustable depth [Fig. 1(f)]. The fiber-bundle itself [Fig. 1(b2)] is composed of 600×600 individual 8  μm-core fibers covering a 2.5×2.5  mm surface, which can guide an image from one end to the other. The fiber elements are patched together by 6×6 along their total length and these patches are assembled only at the extremities of the fiber-bundle, giving it an increased flexibility compared to bundles where all the single fiber elements are glued together along their entire length.

The proximal end of the fiber-bundle is fixed inside a custom designed optical rotary interface [Figs. 1(b3)–1(c3)] (manufactured by JGB SARL), which allows it to rotate relatively to the optical elements (THT Macroscope, SciMedia Ltd) and image sensor [MiCAM Ultima, SciMedia Ltd., 100×100  pixels sensor, 16-bit depth mono, Fig. 1(b4)]. The rotary interface can be finely positioned on a two-dimensional (2D) plane relative to the image sensor with the aid of a 2D translation optic mount [Figs. 1(b5)–1(c5)].

The data from the IMU are transmitted at 50-Hz sampling rate through 4 ultrathin shielded wires (AWG 36 BTA-3607.04 Industrifil France) and an electrical rotary interface [Figs. 1(b6)–1(c6), Jinpat, PT038-0605 - 6 contacts 5 Amperes] to a custom designed controller [Fig. 1(b7), Comprising, PJRC Teensy4.0, Arduino Mega 2560 boards and Trinamic TMC2209 stepper driver].

With the inertial measurements received every 20 ms, the controller computes the absolute orientation of the mouse’s head (yaw component) based on a C++ implementation of the Madgwick orientation filter.^36^ This variable is then used to rotate a stepper motor accordingly [Fig. 1(b8), 42BYGHM809 – 400 physical steps/rev, set at ×8 micro-stepping]. The motor in turn transmits the rotation to the rotary interface by means of a timing belt [Fig. 1(b9)]. The belt is tensioned to minimize mechanical backlash, and is set-up in a 1:3 gear-ratio configuration, increasing output torque to cope with high acceleration rates. A full revolution of the main shaft can thus be subdivided in 9600 individual motor steps, allowing to minimize vibrations and noise generation by smoothing out motion.

### 2.2 Operating Mode

**Fig. 2:**
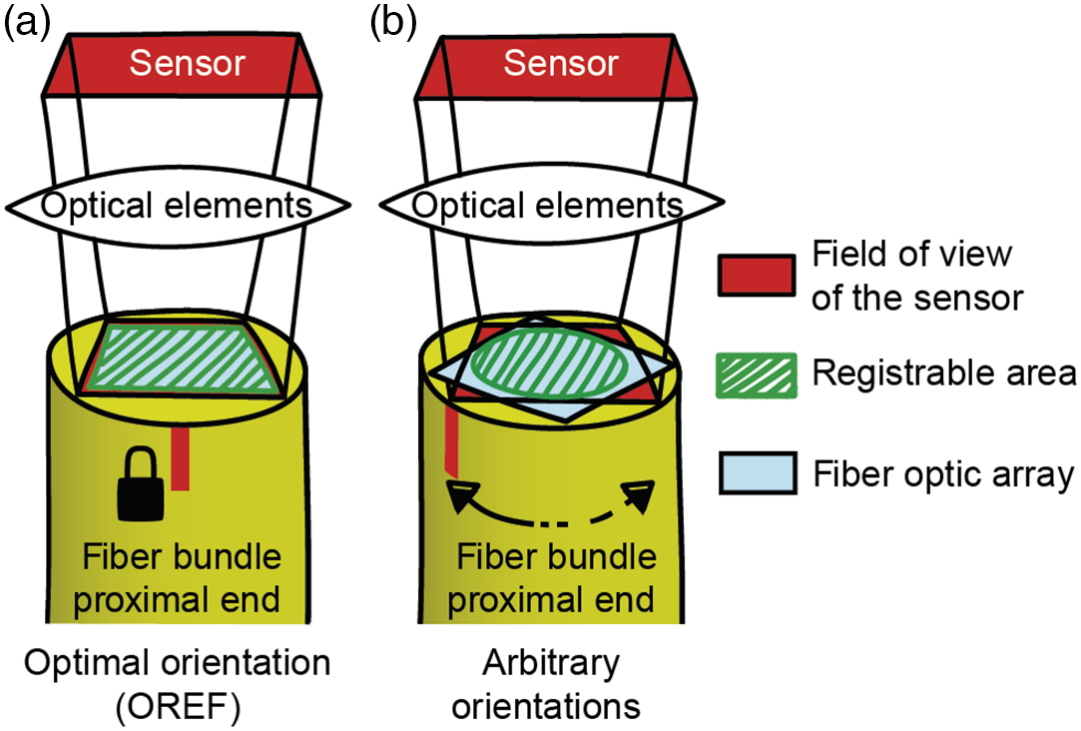
Operating mode of the motorized rotary optical interface. Configuration of the fiber-bundle active area in front of the image sensor at its OREF (a) and at any other non-multiple of 90 deg relative to OREF arbitrary orientation (b). The green hashed area shows the portion of the fiber bundle array (in light blue) that is visible inside the sensor field of view (in red) for the total duration of a sequence of acquisition. With locking in the OREF, this area is larger than with a variable registration orientation, given that the fiber bundle array shape is rectangular.

We have implemented a mode of operation, which ensures acquisition of high-speed and high-quality short sequences of images and allows to compensate for any torsion of the fibroscope due to the mouse rotation between two consecutive sequences of image acquisitions (Fig. 2). We refer to this mode of operation as hardware registration (HR).

Prior to each acquisition, a lock-trigger causes the motor to rapidly reach and maintain an optimal reference orientation (OREF) during the whole sequence. This is the orientation where the largest area of the sensor is filled by the square fiber-bundle imaging field [Fig. 2(a)], thereby ensuring that the largest possible target area and spatial resolution are accessible during the whole sequence. To prevent any inaccuracy in the positioning of the proximal end of the fiber-bundle that could result from mechanical backlash (loss of motion due to coupling tolerances or material elasticity) depending on the direction of motor’s rotation prior to locking, the motor systematically reaches first a position slightly shifted from OREF, for a radial distance greater than the observed backlash, so it then moves to OREF in the same direction prior to each lock.

At the end of the image acquisition sequence (sequence duration depends on user parameters, which were set to 1 second in our case), the rotary joint is released, the motor rotates to match the orientation of the animal’s head as computed from the IMU data, and continues following in real-time its orientation until the next acquisition sequence [Fig. 2(b)].

This mode of operation allows to ensure maximum image stability and to have access to the largest area on the sensor and fiber-bundle elements. It is suitable for acquiring sequences over periods either short enough so that animals do not build significant torsion on the fiber-bundle, or during which the animals are not enticed to turn due to the design of the behavioral task.

### 2.3 Animals

**Fig. 3:**
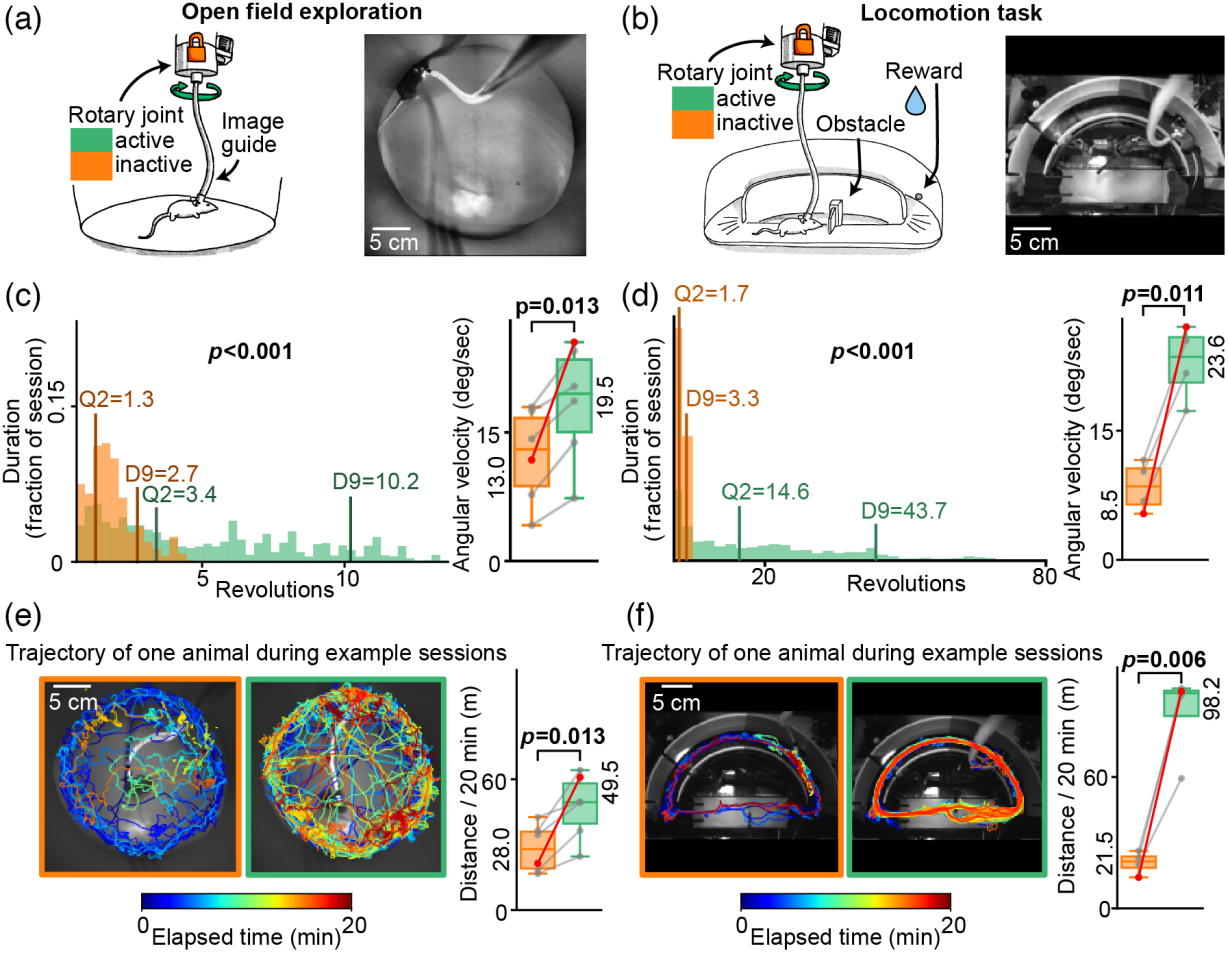
Improvement of the animals’ mobility by the active rotary interface. (**a**) Sketch (left) and snapshot from the infrared video (right) of the open field arena, showing a mouse with a flexible image guide fixed on its head. (**b**) Identical to panel (a), for the locomotion task. All experiments were performed in the dark. For the rest of this figure, the left column panels represent results of open field experiments, and the right column shows results of the rewarded task. Orange colour indicates results from sessions where the optical rotary interface was permanently inactive and green colour, sessions where the optical rotary interface was following the rotation of the animals. (**c**) Quantification of the animals’ rotations during the open field test. Left: distribution [in fraction of the total session time (20 min)] of time spent at different amounts of cumulated rotations, expressed in number of full revolutions (complete 360 deg rotations relative to the starting position, median results for all mice). Vertical lines labelled D9 display the number of revolutions under which falls 90% of the time spent for all sessions, and Q2 display the value under which falls 50% of the time spent for all sessions (median). Right: boxplots showing the mean angular velocity of head rotations over the whole session for each mouse (n=6). Grey lines link values computed from the same animal in active vs inactive joint conditions. The red lines link values from the session pairs exemplified in (e) and (f) (left panels). (**d**) Same as panel (c), for the locomotion task (n=4 mice). (**e**) Quantification of the distance traveled by the animals during the open field test. Left: snapshots from the infrared video taken during behavioral tests, superimposed to mice trajectories, for two 20-min sessions of the same animal. The colour code indicates the time elapsed since the start of the session. Right: boxplots showing the median displacement distance of all mice (n=6). Grey lines link values computed from the same animal in active and inactive joint conditions. The red lines link values extracted from the session pairs illustrated on the left panels. (**f**) Same as panel (e) for the locomotion task (n=4 mice).

Experiments were performed on 8- to 20-week-old C57bl6 mice (Envigo), six males for the behavior experiments (Fig. 3) and one female and two males for the experiments combining cortical imaging and behavior (Figs. 5, 7, and 9). Protocols were in accordance with the French and European (2010/63/UE) legislations relative to the protection of animals used for experimental and other scientific purposes. All experimental procedures comply with the ARRIVE guidelines, and were approved by the French Ministry of Education and Research, after consultation with the ethical committee #59 (authorization number: APAFIS#3561-2016010716016314).

Mice were housed in small groups of siblings (4 to 5 individuals per cage). Housing was enriched with a wheel, a tunnel, nesting material, and toys^37^ in a normal 12-hr light cycle, with food ad libitum.

### 2.4 Surgical Procedure for Head Implant Fixation

Mice were anesthetized with isoflurane (induction 3– to 4%, maintenance 1 to 1.5%). Paw withdrawal, whisker movement and eye-blink reflexes were suppressed. A heating blanket maintained the rectally measured body temperature at 37°C. The eyes were kept moist with Ocry-gel (TVM Lab) and the head was stabilized with a custom-designed nose clamp. Few minutes after subcutaneous injection of lidocaine (4  mg/kg), the skin covering the skull over the dorsal part of the cerebral hemispheres was removed and the skull cleaned.

A removable chamber was then placed over the left hemisphere, filled with warm physiological solution to keep the skull moist and closed with a glass coverslip. Imaging of intrinsic optical signals^1^ evoked by the mechanical stimulation of the right C2 whisker (1 s at 100 Hz) was performed through the intact skull, under 630-nm light, as described in Ferezou et al., (2006).^30^ The reflected light was imaged over the left vibrissal primary somatosensory cortex (vS1) with a MiCAM Ultima camera coupled to a THT Macroscope (SciMedia Ltd). An image taken at 470 nm was then used to locate the activated area with respect of the surface blood vessels. After drying the bone, the head implant could then be fixed, centred on the C2 whisker representation of vS1, with cyanoacrylate glue and dental cement. Mice received a subcutaneous injection of Meloxicam, a non-steroidal anti-inflammatory drug, at 1  mg/kg before anesthesia withdrawal.

### 2.5 Behavioral Protocols

#### 2.5.1 Open field exploration

Mice had unrestricted access to food and water prior to experiments. At the start of each session, they were first attached to the fiber-bundle by their implant and then placed inside an empty 30 cm diameter circular open-field surrounded by 30-cm tall walls, placed under infrared lightning [Fig. 3(a)].

All six mice first underwent one session with the optical interface following mice orientation. The next day, the same six mice underwent a second session with the optical interface remaining static, regardless of their orientation. At the beginning of the session, care was taken to position the fiber-bundle in the least-torsion orientation, before releasing each mouse in the behavioral arena.

The behavior of the animals over the course of the whole session was filmed from the top at 500 Hz with a near infrared camera (BAUMER HXC20) and a high-speed acquisition system (R&D Vision).

#### 2.5.2 Whisker-guided locomotion task in a hemi-circular track

Mice were water-restricted (800  μl per day), and their body weight was monitored daily to ensure a weight >80% of their initial weight before water deprivation.^38^

The apparatus is adapted from Ref. 39 for mice, and consists of a hemi-circular path surrounded by walls, and a straight lane with a length of 28.5 cm and a width of 7.5 cm, along which an “obstacle” is placed randomly either on the right or on the left side [Fig. 3(b)]. The entire arena is devoid of any visible light, but equipped with infrared illumination. Mice can only travel in one direction due to doors automatically closing after their passage. Each time mice complete the full path, they receive a reward (one 8  μl drop of water). This motivates mice to run continuously during the session.

The same 6 mice that previously accomplished the open field exploration first performed 14 training sessions without the fiber-bundle (1 session per working day, ending either after 20 minutes or 100 reward deliveries), during which they were able to familiarize themselves with the apparatus and its functioning. Out of these six mice, five have learned to run in the maze to receive water rewards.

These five mice then underwent two sessions, during 2 consecutive days, where the fiber-bundle was attached to their head and the optical rotary interface was active and followed their rotation. The next 2 days, they all underwent two more experiments with the fiber-bundle attached again but the optical interface staying static throughout the whole session regardless of the orientation of the animal. Care was taken at the beginning of the sessions to orient the fiber in the least torsion orientation as described for open-field experiments. We recorded the behavior of the animals in the exact same manner as for open-field experiments. To characterize the effect of joint activation on the mice behavior, we compared the first session with the rotary interface activated versus the first session with the rotary interface inactive. As we accidently lost the IMU data from one animal, data from four mice could be analyzed.

### 2.6 Optomechanical Characterization Experiments

**Fig. 4:**
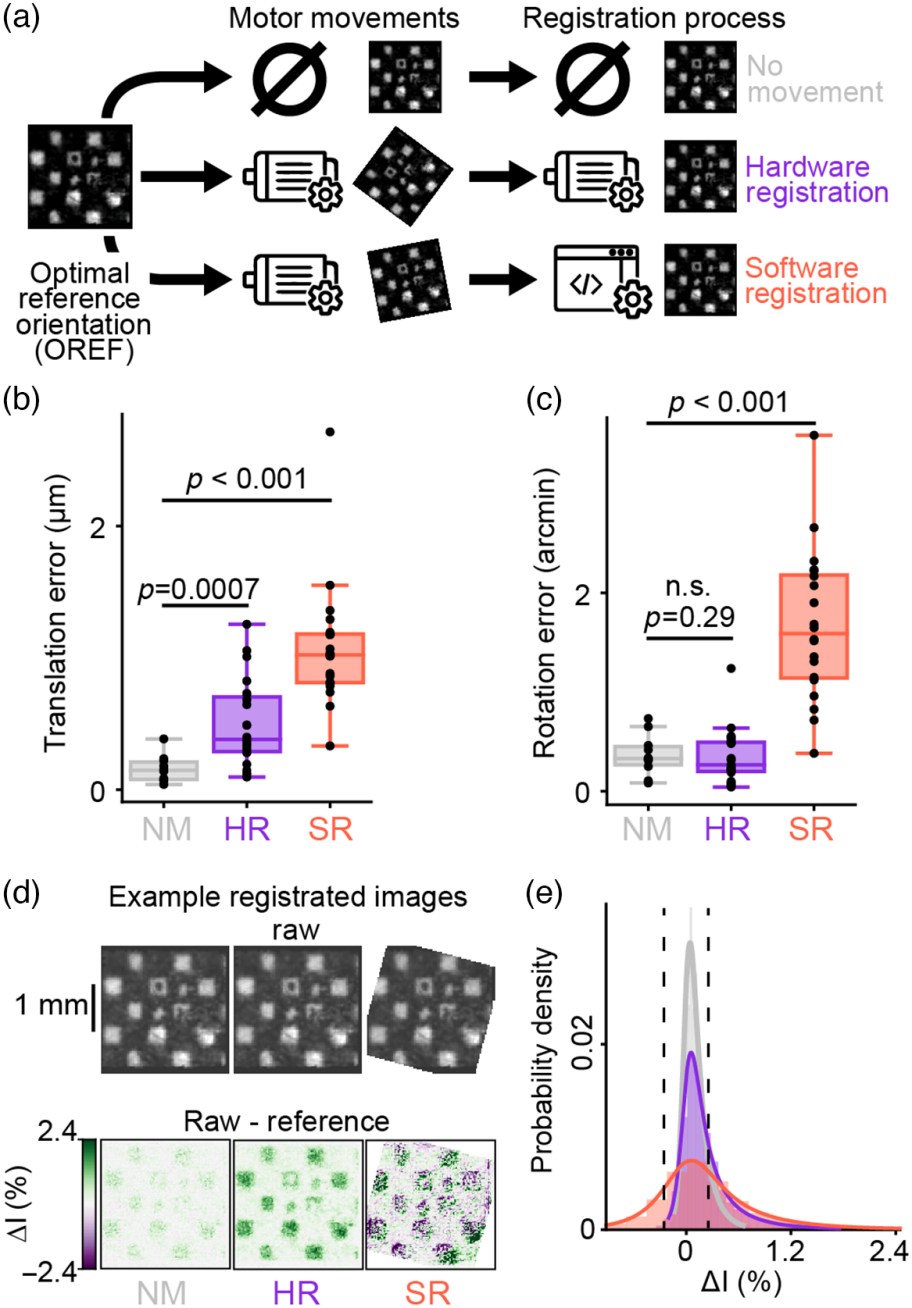
Locking the interface at a reference orientation in front of the sensor produces best optical performances. (**a**) Illustration of the different steps carried out to evaluate the accuracy of the hardware (HR) and software (SR) registration methods. A reference image was first acquired at OREF. A series of 25 images was then captured without moving the interface in-between [no movement (NM) condition]. A second set of 25 images (HR) was taken with the interface in the reference position, but with the rotating interface being moved to an arbitrary orientation (between 9 deg and 345 deg) between each image acquisition. A third set of images (SR) was captured at orientations between 9 deg and 345 deg and registered off-line to the initial reference image, based on the known motor position (see methods). (**b**) Translation error and (**c**) rotation error from frames of NM, HR and SR sequences compared to the reference image. These are the results of an affine transformation estimation based on pairs of tracked points on reference and test images (see methods). (**d**) Example raw images registered with different methods (top) and their difference with reference image (variation of pixel intensity (ΔI) expressed in percentage of full pixel intensity range, bottom). (**e**) Probability density graph of the variation of intensity between each pixel of the registered image, and its equally located counterpart on the reference image, for the two methods of registration and the control condition (n=25 for each group). Fainted histograms represent data distributions, curves represent normal inverse Gaussian fits of the data distributions. Black dashed-lines indicate ±0.25% intensity variation compared to the reference image.

To characterize the mechanical accuracy and the resulting optical reproducibility that our system yields in different conditions as well as background noise, we acquired three types of image series. The first type comprises images captured at OREF with no movement of the rotary interface in between each image. It is referred as the “no moving” condition [NM, Fig. 4(a) Top]. The second type was acquired by implementing the HR mode of operation as described in Sec. 2.2, each image sequence was acquired at OREF, but the rotary interface made series of displacement from 9 deg to 345 deg in between each sequence. In this case, only the 125’th frame of each sequence of 256 frames was used for the registration measurements, as we intended to evaluate the impact of the motor movement (and during a sequence there is none). This is referred to as the HR condition [Fig. 4(a) middle]. The last type of image series consisted in acquiring consecutive images with the rotary interface positioned at different locations ranging from 9 deg to 345 deg relative to the OREF. Each image was saved along with its corresponding motor orientation, allowing for offline software registration (SR) with OREF (as described in Sec. 2.8). Hence, this condition is referred to as SR [Fig. 4(a) bottom].

### 2.7 VSD Imaging in Freely Moving Mice

#### 2.7.1 Surgical procedure and dye staining

After being trained in the whisker-guided locomotion task (as described in Sec. 2.5.2) without and then with the fiber-bundle attached to their head implant, mice were anesthetized with isoflurane (induction 3 to 4%, maintenance 1 to 1.5%). After verification of reflex suppression, a craniotomy and durectomy were then performed inside their head-fixation implant, as described in Ref. 30. The implant features openings on the sides to ease the access of surgical tools to the skull and dura mater. To minimize the time spent under anesthesia and ensure a fast recovery, this intervention was performed as quickly as possible. Overall, the anesthesia duration ranged from 40 to 90 min. Just before withdrawal of anesthesia, mice received a subcutaneous injection of Meloxicam 1  mg/kg, and we added a custom designed chamber inside the implant to hermetically contain a volume of about 200  μl of the VSD RH1691 (1  mg/ml, Optical Imaging Ltd.) on top of the exposed cortex. Animals were then allowed to recover from anesthesia during the 1 to 2 hrs needed to stain the cortex with the dye. As soon as they showed an active behavior indicative of a good recovery from anesthesia, we head-fixed the mice by their implant to carefully open the chamber and wash out the dye over the cortex. Finally, the fiber-bundle was carefully brought down and rigidly fixed to the implant, being in direct contact with the stained cortex. The mice were then placed in the hemi-circular track, and ready to start the recording session.

#### 2.7.2 VSD imaging during the whisker-guided locomotion task

During the functional imaging session, in the same way as during the previous training sessions, the mice received a reward for each complete run in the hemi-circular arena, which is hereafter referred to as a trial. As soon as a mouse crossed the infrared beam located before the beginning of the straight lane of the maze, it triggered the rotary interface to reach OREF, as described in Sec. 2.2. Once OREF was reached, the illumination of the cortex through the fiber-bundle started. The 630-nm excitation light from a 100 W halogen lamp was reflected using a 650-nm dichroic mirror within the THT Macroscope and focused onto the proximal fiber end. A second infrared beam located at the beginning of the straight lane next triggered the acquisition of a ∼1 second sequence of 512 images collected at 2-ms sampling interval and 2-ms exposure time by the Micam Ultima camera. The illumination was stopped, either at the end of the acquisition sequence (resulting in a maximum of 3-s total illumination time), or in case the mouse failed to cross the acquisition trigger beam in less than 2 s, and the optical interface started following mouse orientation again. This allowed to avoid bleaching the dye while no acquisition was ongoing. The preacquisition illumination period helped to avoid acquiring signal in the steeper phase of the VSD fluorescence bleaching effect (see Appendix A, Fig. 6 for more details about the photobleaching and its correction).

#### 2.7.3 High-speed behavioral videos

Synchronously to the acquisition of cortical fluorescent signals, behavioral activity was filmed with an infrared camera (BAUMER HXC20), at the same 2-ms sample interval for the same duration of 512 frames (R&D Vision). High-contrast images were obtained by placing an infrared backlight under the transparent floor of the straight lane of the maze (850 nm, custom designed).

#### 2.7.4 Anatomo-functional mapping of vS1

At the end of the behavioral training session, without detaching the fiber-bundle distal end, we fixed animals by their head implant, anesthetized them with Isoflurane (induction 3 to 4%, maintenance 1 to 1.5%), and checked for reflex suppression. We then imaged fluorescent signals evoked by the deflection of 2 to 5 different whiskers stimulated individually to map their cortical representation within vS1, as described in Hubatz et al., (2020),^40^ and illustrated in Appendix B [Figs. 7 and 9(c)]. After this process, mice were deeply anesthetized with sodium pentobarbital (140  mg/kg) and perfused intracardially with saline followed by paraformaldehyde (4% in 0.1 M phosphate buffer). After an overnight postfixation in paraformaldehyde, the brains were cut in 100-μm-thick tangential sections and stained for cytochrome oxidase. As described in Perronnet et al., (2016),^41^ the layer 4 barrel map was then reconstructed from the stained histological slices, and aligned to the previously acquired functional VSD images using the superficial blood vessels as anatomical landmarks.

### 2.8 Data Analyses

#### 2.8.1 Animal mobility quantification

The animals’ positions on the behavioral videos were tracked frame by frame by means of the DeepLabCut toolbox.^42^ The feature detector deep learning algorithm was trained to identify two markers placed on the animals’ head implant, their nose tip, and the base of their tail. Epochs where the nose tip or one of the two markers were not extracted with confidence by the DeepLabCut toolbox were removed from the analysis.

To follow the rotation of animals, we used the yaw component of the output of the orientation filter algorithm of the IMU that was saved during experiments.

#### 2.8.2 Estimation of spatial transformation between images

For the measure of the displacement between two images, we used a scale invariant feature transform algorithm (SIFT^43^), which identifies the best features to track in both images and match them. Matched features were then filtered with the RANdom SAmple Consensus (RANSAC^44^) method to find the best affine transformation that explained the uniform image rotation and translation.

#### 2.8.3 Software image registration (SR)

The algorithm implemented to register images via software was developed to be compatible with real time imaging and to be independent of the content of the image, using the rotation of the motor driving the rotary joint as a single entry for each frame.

Mechanical inaccuracies in the positioning of the fiber-bundle proximal end inside the rotating shaft can cause the center of the image to describe a circle, instead of a point, during the rotation of the interface in front of the image sensor. To correct for these reproducible inaccuracies, we simply used a calibrated look up table (LUT) of translation and rotation offsets. We built this table by first capturing a frame at the OREF and then capturing images after positioning the motor at given intervals relative to the OREF. Transformations between images were then computed using the SIFT-RANSAC method described in the section above, and these rotation and translation offsets were used as the LUT entries for each motor orientation.

In the end, the software registered images were obtained by feeding the motor orientation into the software. The LUT resulting from the calibration procedure was then used to compute discrete subpixel values of the rotated frame with a cubic interpolation performed in one step to minimize image blurring with multiple consecutive interpolations.

#### 2.8.4 Processing of voltage sensitive dye images

Variations of fluorescence over time were computed pixel by pixel as $\Delta F/F_{0}$, $F_{0}$ being computed as the mean of three frames around a chosen reference frame (specified below, in the results section).

To correct for bleaching related artefact, for each image sequence, a 2.5-Hz lowpass Butterworth filter was applied to the profile of fluorescence computed over a large region of interest. The second degree polynomial fit of this filtered trace was subsequently subtracted from the original image sequence (as described in Appendix A, Fig. 6). A 2D gaussian filter (5×5  pixels) was used in some cases to reduce spatial noise on the images.

An inverted fast Fourier transform was used to correct for a visible spatially patterned noise, most likely due a moiré effect linked to the visible spatial structure of our fiber-bundle and its relative orientation in front of the sensor (see Appendix C, Fig. 8).

## 3 Results

With the aim of enlarging the spectrum of behavioral tasks compatible with fibroscopic optical methods for reading and controlling neuronal activity in mice, we have developed a motorized rotating optical interface. In its principle, this active optical commutator is adaptable to any type of fibroscopic approach, but here we have implemented and validated it for VSD imaging of a large field (2.5×2.5  mm) of the cortical surface through a 90-cm-long flexible fiber optic bundle.

### 3.1 Mobility Measured with Two Different Behavioral Tests

To evaluate the benefit that the active optical joint represents in terms of behavior, we quantified mice displacements when the rotation of the proximal part of the fiber-bundle was fixed, compared to when it actively rotated to follow their orientation. We performed this comparison in the context of an open field test [Fig. 3(a)], but also while mice were performing a whisker-guided locomotion task [Fig. 3(b)]. In this operant conditioning task, water-deprived animals receive a water-reward each time they complete a full path in a hemi-circular arena (see Sec. 2.5).

To assess to what extend the optical joint could restore the ability of mice to rotate during these free exploration and goal-directed behavior contexts, we first computed the distribution of the time spent at different amounts of cumulated rotations [Figs. 3(c) and 3(d), left panels], expressed in full revolutions relative to the orientation at the start of the session, where the torsion was minimal. A full revolution being here defined as a complete 360 deg rotation of the mouse, which could be achieved either by taking a large circular path in the behavioral arena, or through a simple rotation around its axis at a given location. Having the rotary joint active allowed mice to make significantly more consecutive turns in the same direction compared to the inactive joint condition, in both experimental conditions (P<0.001, Wilcoxon signed-rank test). Overall, in the open field test, mice spend 90% of their time below 2.7 full revolutions from their initial position when the joint was inactive, but up 10.2 revolutions while it was active. During the locomotion task, they spent 90% of their time below 3.3 revolutions with the inactive joint but up to 43.7 revolutions when the joint was active. The benefit from the rotary joint was indeed accentuated in the locomotion task where animals, rewarded at each lap of the hemi-circular track, were strongly enticed to cumulate rotations. Nonetheless, while the joint was inactive, the range in which they would spend 90% of their time was similar for both tasks. This suggests that the approximate maximum torsion they could overcome given their strength and the flexibility of our image bundle fell between 3 and 4 turns.

To overcome this limitation of their motion while the joint was inactive, mice could have used a strategy consisting of revolving around themselves to lower the torsion of the image guide when it became too high, and then continue moving in the initially intended direction. This strategy could represent an advantage specifically in the context of the goal-directed locomotion task were animals were motivated to turn repeatedly in the same direction. To assess this possibility, we measured the mean angular velocity for each experimental condition [Figs. 3(c) and 3(d), right panels]. We observed that in contradiction with this hypothesis, the angular velocity was lower for the locomotion task than in the exploratory open field condition. These results further show that the angular velocity significantly increased with the activation of the joint in both contexts (P=0.013 and P=0.011, for the open field and locomotion task conditions, respectively, paired T-test). Note that this increase was much steeper in the context of the locomotion task (169% versus 50% for the open field condition).

With the aim of evaluating how the rotary joint, by limiting the torsion of the image guide, could impact the overall displacement of the animals, we next analysed the trajectories of the mice during all behavioral sessions.

Left panels in Figs. 3(e) and 3(f) illustrate the trajectories of the same representative animal along two sessions; with or without the joint active, for the open field test [Fig. 3(e)], and for the locomotion task [Fig. 3(f)]. In both contexts, this particular mouse showed a tendency, after a few minutes, to remain almost static for prolonged periods of time when the joint was inactive. However, when the joint was active, the animal travelled across a much greater area, and explored the behavioural arenas more evenly. One can note that in the locomotion task, when the joint was inactive, the location at which this mouse restricted its displacements for prolonged periods of time was at the vicinity of the reward area.

The overall effect of the rotary joint on animals’ trajectories was quantified by computing the mean displacement distance during each session for all mice [Figs. 3(e) and 3(f), right panels]. We observed that the activation of the joint significantly increased the mean animals’ displacement distances in both contexts (P=0.013 and P=0.006 for the open field and locomotion task conditions, respectively, paired T-test). Once again, while the effect is significant in the open field context, it is much larger in the rewarded task (327% increase versus 68% for the open field task).

These results indicate that in a classical fibroscopic configuration, the torsion of the fiber-bundle induces fatigue and/or pain that reduce not only the rotational movements but also the overall displacements of the animals. The motorized rotary joint indeed significantly improves animal mobility, even during a simple exploratory behavior in an open field arena.

This improvement in mobility, together with the ability to follow continuous rotations, offered by the active rotary joint undoubtedly broadens the spectrum of freely moving behaviors compatible with fibroscopic methods.

### 3.2 Accuracy of the Rotary Interface in Different Operating Modes

To acquire registered sequences of images with fibroscopic imaging, here we used a mode of operation where we can briefly lock the rotation of the proximal end of the fiber-bundle in front of the sensor in an OREF during short sequences of image acquisition, as described in Sec. 2.2 (Fig. 2). This HR mode is particularly well suited for high-speed optical methods such as VSD imaging, for which it is not desirable to make continuous recordings (to prevent photo-damage of the tissue). With this strategy, the image stability inside a sequence is very high. However, the accuracy of the repositioning of the rotary joint in front of the sensor before each sequence is an important requirement to allow analysis of the signal originating from defined spatially located areas of the tissue across several sequences or trials.

Another possible mode of operation of the optical joint, which would be particularly well suited for long continuous recordings at lower sampling frequency (as for imaging with calcium indicators), could be to let the motor follow the animal’s orientation in between each single frame acquisition, and lock it briefly only during each frame’s exposure time. The motor position corresponding to each acquired frame is saved synchronously with the image. In this mode of operation, named here SR, registration of the images is computed by leveraging the saved motor orientation and not from an estimation of image rotation based on spatial features in the signal (as described in Sec. 2.8).

We evaluated the accuracy of image registration achieved with HR and SR modes of operation of the optical joint, and compared them to a control situation where no movement occurs between two acquired images (NM) as an ideal registration situation, as described in Sec. 2.6.1 [Fig. 4(a)].

Translation error after HR proved to be very reliable, with a median value of 0.38  μm. [Fig. 4(b)]. Surprisingly, the SR registration method yielded quite similar results, with median translation error of 1.02  μm. Both were significantly different from the control ideal registration case (NM), with which the median translation error was 0.14  μm. Here this value is assumed to result mostly from the imprecision of the optical measure of translation error, different from 0 due to the slight background noise in the optical signal.

The translation error of the HR method overall remains below 1  μm. For reference, one pixel of our sensor corresponds to a sampled square of 25  μm size. Thus, a translation error of only 1  μm implies that depending on the direction of that translation, from 4% and up to 5.6% of the sampled area would be biased by light rays that would otherwise have been received by neighbors’ pixels in the reference frame.

For rotation error, results were even better for the HR method, which did not differ from the control NM condition [Fig. 4(c)]. The SR method, however, suffered from a greater variability in registration, although it remained very precise with a median below 2 arcmin of error (NM: 0.32, HR: 0.26, SR: 1.59 arcmin).

This spread in the efficacy of registration was probably not due to motor or mechanic imprecision, as HR at OREF is very precise, but rather to an imperfectly calibrated offset rotation LUT (described in Sec. 2.8 and discussed in Sec. 4.2) that changes at the different orientations of the motor.

We then quantified how such spatial imprecisions in the registration erroneously impacted pixels’ intensity values relative to the reference image. Examples of raw and relative variations of pixel intensity values with the two registration methods and the control condition show at first sight that SR registration suffers from higher errors [Fig. 4(d)]. This may be caused by the nature of the SR technique, which interpolates subpixel values with bi-cubic fitting. This inherently results in errors compared to the local distributions of real pixel values, rather than due to a higher degree of rotation and translation imprecision. Probability density functions describing the distribution of the error in pixel intensity values for the two registration methods and the control condition are shown in Fig. 4(e). These results demonstrate that the probability for a pixel to have 0.25% (of the full pixel values range) or less variation compared to its identically located pixel in the reference image is 63% for NM, 43% for HR, and 22% for SR.

Overall, while both registration methods are significantly different from control, the hardware and SR methods show levels of spatial precision as well as intensity value changes that are compatible with one photon imaging techniques.

### 3.3 Example of Application: Linking VSD Imaging of Cortical Dynamics to Behavior at 500 Hz During a Whisker-Guided Locomotion Task

**Fig. 5:**
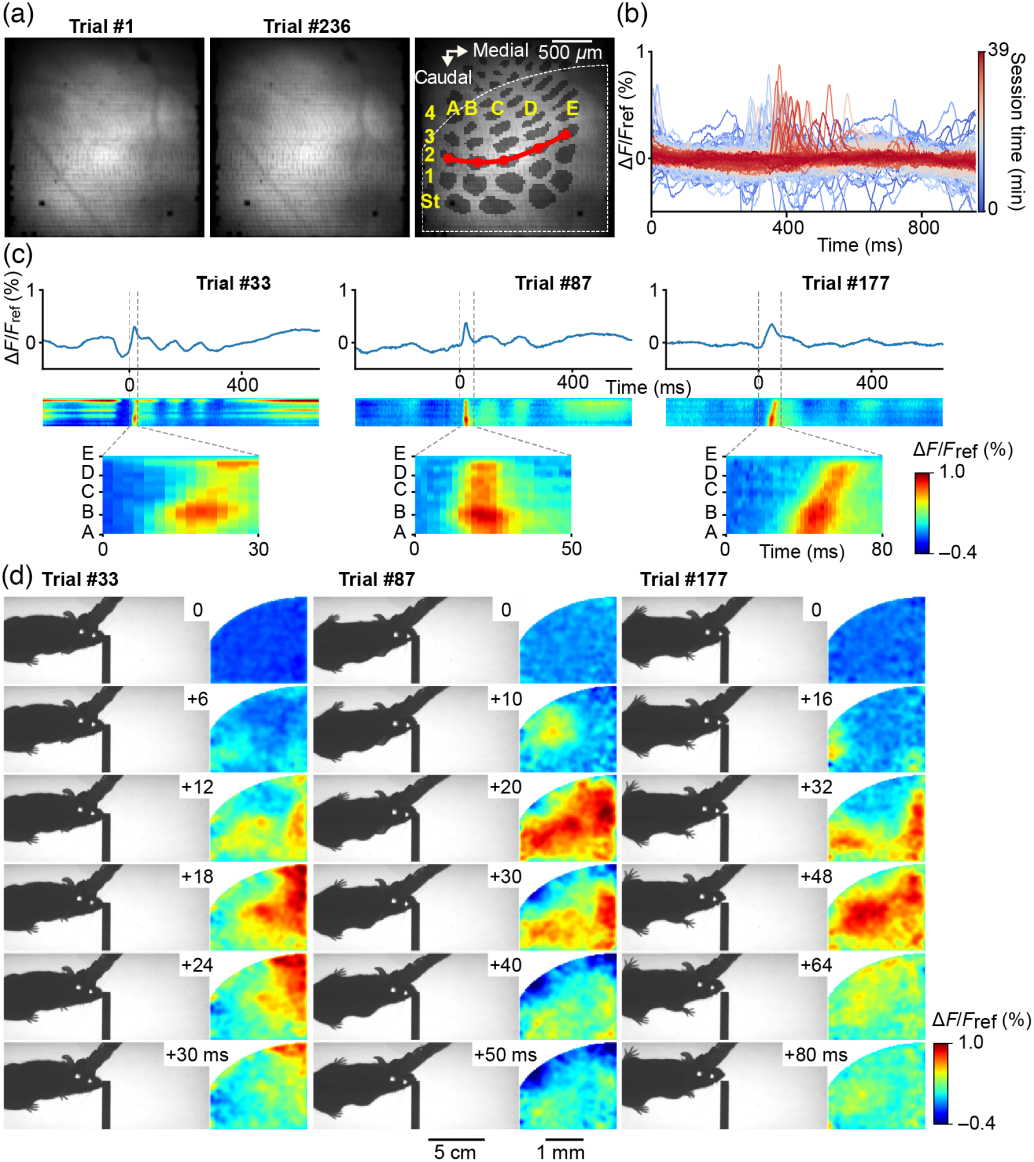
Linking VSD imaging of cortical dynamics to behavior at 500 Hz during a locomotion task. (**a**) Resting VSD fluorescence imaged through the fiber-bundle at OREF at the start (trial #1, left), and the end (trial #236, middle), of the session of recording during the locomotion task. The layer 4 barrel map of vS1 reconstructed from a post hoc cytochrome oxidase staining is overlaid on the image (darker gray). Rows are named with letters and arcs with numbers, except the more caudal arc that corresponds to the four straddlers (St). Part of the field of view used for subsequent fluorescence quantification is shown as dashed white outline. The red line through arc 2 was used to compute the linescan plots shown in panel (c). The two dark spots, visible at the bottom-left of the field of view and inside alpha barrel, are broken fiber subassemblies (6×6 8-μm fibers). (**b**) Variation of VSD fluorescence quantified from a large region of interest [dashed white outline in panel (a)] for all the trials of this session (from the first trial in dark blue, to the last trial in dark red). A large proportion of trials exhibit large cortical depolarization at around 300 to 600 ms from trial onset. (**c**) Fluorescence profiles of three example trials from the same session (top) and corresponding linescan plots (middle, time on x axis and space on y axis) computed from a line spanning the second arc of the barrel map [red line in panel (a)]. Reference time 0 corresponds to the onset of a large cortical depolarisation displayed with expanded scale just below (bottom). (**d**) For each trial, on the right are shown 6 snapshots showing relative variations of fluorescence ($\Delta F/F_{\mathrm{ref}}$, $F_{\mathrm{ref}}$ being an average of three frames centered at time 0). On the left are the corresponding snapshots of the infrared behavior video, captured synchronously with the VSD images, in the straight lane of the track.

Initially, we developed this active rotary interface for the visualization of the mesoscopic scale dynamics over the whiskers’ representation in the primary somatosensory cortex (vS1) of mice engaged in a whisker-guided locomotion task that involves multiple rotations of animals running along a hemi-circular track. With this purpose, we used VSD imaging, which correlates with changes in membrane potential of layer 2/3 neurons at the millisecond timescale.^30^ In our experiments carried out under infrared light, the behavior of the mice was recorded synchronously with the cortical activity, at 500 Hz, while mice ran in the straight lane of the previously described hemi-circular track. An obstacle was positioned randomly on the right or the left side of this straight lane, so the mice had to actively probe their environment with their whiskers to avoid collisions. Figure 5 illustrates an example experiment (see also Appendix D, Fig. 9, for additional examples). During this session, the gain of mobility provided by the active rotary interface allowed us to image cortical activity during up to 39 min, and 236 rewarded full laps of the hemi-circular track (called here trials) with stable signal over time [Figs. 5(a) and 5(b)]. The VSD resting fluorescence images taken at OREF on the very first and very last trials of the session reveal the repositioning accuracy of the joint throughout the session, and the stability of the optical access to the cortex [Fig. 5(a)]. One can note that the pattern of multiple 6×6 multi-fiber elements of our image guide is visible on these images (Sec. 2.1, Ref. 30). vS1 is characterized by the presence, at the level of the layer 4, of cellular aggregates, named barrels, which are topologically organized as the whiskers on the animal’s snout. This barrel map could be reconstructed post-hoc from tangential brain slices stained for cytochrome oxidase, and realigned with the functional images [Fig. 5(a), see details in Sec. 2.7, Ref. 41].

Most trials exhibited large wave-like activities recorded consistently from the beginning to the end of the session [Figs. 5(b), 9(a), and 9(b)]. Indeed, large static or traveling depolarizations occurred in a high proportion of the trials. These events frequently occurred 300 to 600 ms following the onset of the acquisition, while the mouse was in the vicinity of the obstacle, in the central portion of the straight lane. Although the detailed description of these events and their relation to the animals’ behavior is out of the scope of this study, it can be noted here that they appeared to have variable spatial origins, and directions of propagation, as exemplified by three selected trials [Figs. 5(c) and 5(d), see Appendix D, Figs. 9(a) and 9(b) for additional examples from other experiments]. Few trials showed activity emerging in the barrel cortex, as for trial #87. Other events seemed to emerge simultaneously in multiple areas, inside or outside the whiskers’ representation field, as in trials #33 and #177. Note that the visualization of absolute fluorescence signals during these same trials demonstrates the stability of the images despite the animal running in the behavioral arena (see Appendix E, Fig. 10).

Overall, we demonstrated that the motorized optical rotary joint allows to make repeated acquisitions of cortical activity on freely moving animals over long periods of time, with good signal levels, similar to those shown in a previous study carried on a similar setup without optical rotary joint.^30^ However, the strong improvement of the animal’s mobility and ability to turn provided by the joint gives the opportunity to couple such recordings with a much broader range of behaviors.

## 4 Discussion

To allow the combination of fibroscopic approaches for the imaging or manipulation of neuronal activity in free-moving mice with a broad spectrum of behavioral protocols, we have designed and implemented an active optical rotary joint, which can follow the rotation of the animal by means of an embedded inertial measurement unit. We have established that its use strongly enhances the animal mobility in different behavioral contexts, and advantageously opens the possibility of using fibroscopy during a task that requires the animal to rotate continuously in the same direction. We indeed demonstrated its effectiveness for VSD imaging of cortical activity in vS1 of mice engaged in a whisker-guided locomotion task on a hemi-circular track. Moreover, the different possible modes of operation of the rotary joint make it adaptable to the needs of various optical methods and behavioral tasks.

### 4.1 Possible Modes of Operation of the Rotary Interface

The HR mode that we first implemented is well suited for short sequences of imaging/photo-stimulation at high sampling rates, alternating with periods of free behavior, exempt of optical acquisition/stimulation. By blocking the fiber-bundle proximal end at OREF during entire series of acquisitions/stimulations, this sequential mode of operation optimizes the spatio-temporal resolution and quality of optical signals.

In the second operating mode of the rotary interface, the motor is never locked at OREF, but follows the animal’s orientation in a semi-continuous manner. It is indeed locked briefly at arbitrary rotation angles only during each frame’s exposure time to prevent motion blur, and is free to follow the animal’s orientation in between each frame. This mode of operation implies that photon collection has to be spaced by at least the amount of time necessary for the motor to move the average angle animals can rotate in the interval between two frames. It is therefore suitable for imaging at lower sampling rates, as for calcium imaging. Images are then registered via software means, based on the motor position saved at the time they were captured. This approach thus has the advantage of allowing for very long acquisitions, but does so at the expense of spatio-temporal resolution. In addition, it should be noted that in this mode, the illumination homogeneity will matter more than for the sequential mode. Indeed, as here the illumination system remains static, while the orientation of the fiber-bundle proximal end is not, inhomogeneous light source could induce variations of excitation light likely to impact emitted fluorescence signals.

Finally, the rotary interface could also be used in a third, hybrid mode, for which the fiber-bundle proximal end would be locked at OREF and the mouse orientation constantly monitored by means of the inertial measurement unit, as for the first sequential mode. As soon as the offset between OREF and the animal’s orientation exceeds 360 deg, fast movement of the motor could be triggered to compensate the torsion of the image guide. The motor would then quickly lock its proximal end back at OREF. This cycle could continue indefinitely, resulting in a pseudocontinuous acquisition. In terms of optical properties, the functioning of this mode of operation is similar to the first sequential mode of operation and should give exactly identical results, optimizing optical quality of the signal, but would result in short interruptions occurring at unpredictable times (depending on the behavioral task design).

### 4.2 Mechanical Factors that may Impact the Registration Accuracy

The motorized rotating optical interface could be impacted by a mechanical backlash phenomenon. Backlash is here defined as a loss of motion occurring because the change in direction of the motor is not applied instantaneously to the mechanically coupled part, due to coupling tolerances or material elasticity. It can be minimized but not removed entirely, unless using specific, expensive so-called “zero-backlash” mechanical systems. In the implementation of the HR mode of operation of the rotary interface, we easily circumvented this problem by first reaching a slightly shifted position relative to OREF for a radial distance greater than the observed backlash. The OREF position was next consistently reached through a rotation in the same direction prior to each lock.

In contrast, in the SR mode of operation intended for continuous recording, since this should be done between the acquisition of each frame, movements will require to be more time efficient and this solution may not easily be applicable, in which case a greater backlash limited mechanical system should be required. Electromechanical inaccuracies in the positioning of the fiber-bundle due to backlash or fast movements could also be corrected with a closed loop control system, comprising a rotary encoder fixed at the fiber-axis preferably to the motor axis.

Another important mechanical aspect, especially for working in a continuous acquisition mode, is the axial alignment between the center of the sensor and the central axis of the fiber-bundle proximal end. As described in the method section, this can be addressed on a software basis, by calibrating a LUT of rotation and translation offsets based on motor position relative to OREF. It could also be solved by specific mechanical designs ensuring high levels of concentricity such as concentric clamping systems (i.e., collet chucks). However, for our specific use-case, imaging the whole vS1 cortex with subcolumnar resolution (25  μm per pixel), the level of spatial accuracy with HR at OREF was more than sufficient by simply using three screws axially placed 120 deg apart to adjust the concentricity of the rotary joint.

### 4.3 Remarks for Inertial Measurement-Based Orientation Estimation

To our knowledge, no motorized rotating joint allowing the transmission of a coherent image have been developed before. Active rotative joints have been implemented only for photometry and electrophysiology or to transmit power to, and data from, miniaturized head mounted microscopes. All these systems are based on a measure taken on the joint itself, by means of either a torque sensor (see torque sensor assisted rotary joints commercialized by Doric or a hall effect sensor (as within the FinchScope project^45^). They therefore rely on an indirect measurement of the animal’s rotation, which is made possible due to the torsion of the cable link between the animal and the joint. These approaches are therefore highly dependent on the flexibility properties of this cable, thus requiring sensitivity adjustments to work well on different animals with different fiber-bundle types. This sensitivity issue simply does not exist with the use of an on-board IMU that allows the acquisition of a direct measure of the fiber-bundle orientation, regardless of its flexibility properties (with the very small constraint of having four additional thin wires following the image guide).

On the other hand, the absolute orientation calculation heavily relies on magnetometer measurements. It is the only component of the three integrated devices (accelerometer, gyroscope, and magnetometer) that does not measure intrinsic motion but an extrinsic reference, and as such, that does not suffer from error accumulation. Nevertheless, this component is sensitive to magnetic distortions and requires calibration. If imperfectly done, it results in loss of accuracy of the orientation calculation.

Multiple algorithmic strategies have been recently proposed to better generalize the orientation calculation regardless of environmental perturbations,^46^ or calibration specificities, using deep neural networks instead of more generic algorithms,^47^ which could make this approach easier to set up.

### 4.4 Using Optical Means to Compensate for Image Rotation: An Interesting but Not Yet Practicable Approach

To perform continuous imaging without the need to perform mechanical rotation stops during exposure time of an image, a purely optical method to straighten up the optical flow in real time could be considered. This is indeed theoretically achievable using a rotating dove or other prisms (for review, see Ref. 48) that in general terms, produce a rotated image by twice the amount of rotation of the prism. They, however, suffer from a major drawback for our current application, being that “to rotate the beam without any associated translation or angular deviation, the axis of the Dove prism must be aligned to within a fraction of the wavelength of the light.”^49^^,^^50^ For wavelengths used in fluorescence excitation in neurophysiology, this corresponds to micrometric precision. While this is already a challenge in a static design allowing the correction of a single orientation, correcting for a tunable orientation requires the prism to be mounted in a mechanical rotating device, whose precision must also be micrometric. Such optical-mechanical methods would thus be extremely difficult to implement in practice.

Other techniques allowing non-mechanical image rotation have been developed,^51^ but constrain the correction of the rotation to discrete orientations. This would thus not alleviate the need for making stops during exposure times of single images for continuous acquisition.

Finally, one recent article proposed a—still theoretical—scheme to allow such continuous correction of an arbitrary rotation in a non-mechanical fashion.^49^ However, the authors mention that the rotation with this technique is wavelength sensitive, and thus not well suited for fluorescence imaging where several wavelengths are transmitted bidirectionally.

Overall, for the specific use of imaging small fluorescence changes with low artefactual perturbations, all the solutions based on purely optical means to strengthen up a rotating image do not appear to provide viable solutions so far.

### 4.5 Methodological Outlook

Convinced of the need to combine optical methods for measuring and monitoring neural activity with behavior in the awake animal, the neuroscientific community has made considerable efforts, either to adapt behavioral task designs to be compatible with animal’s head fixation under the most powerful optical devices, or to adapt optical methods for a use in freely moving animals. Alternative approaches that use a fiber optic bundle to link a powerful and potentially cumbersome optical system to a free-moving animal have so far been hampered by the fact that the problem of optical cables twisting with animal’s rotations cannot be handled as easily as for electrical cables.

The motorized rotating interface we propose here, solving this limitation due to optical cable torsion in an efficient and relatively easy to set up manner, opens up a new field of possibilities for fibroscopic approaches. It indeed allows combining the use of any optical device for imaging (or multisite photometry^33^^,^^34^), and/or patterned photoactivation, with a large range of freely moving behaviors, including any operant conditioning task requiring the animal to rotate continuously in the same direction. The exploratory behavior of the animals observed here in the open-field condition suggests that the rotary joint represents a considerable asset for studying other spontaneous behaviors such as social interactions. Of course, as soon as one wishes to record several animals simultaneously, problems of cable entanglement would arise, which could be solved only with a completely wireless approach. Although such technologies are emerging for one-photon endoscopy, allowing either onboard data acquisition^52^^,^^53^ or radiofrequency data transmission,^45^^,^^54^^,^^55^ they are relatively cumbersome for mice due to the necessary integrated battery, and their temporal resolution remains limited (below 50 Hz).

Overall, fibroscopic approaches combined with an optical rotary joint represent an outstanding tool to link neuronal activity with behavior in mice, at the millisecond timescale.

## 5 Appendices

### 5.1 Appendix A: Photobleaching of the VSD Fluorescence and its Offline Correction

**Fig. 6:**
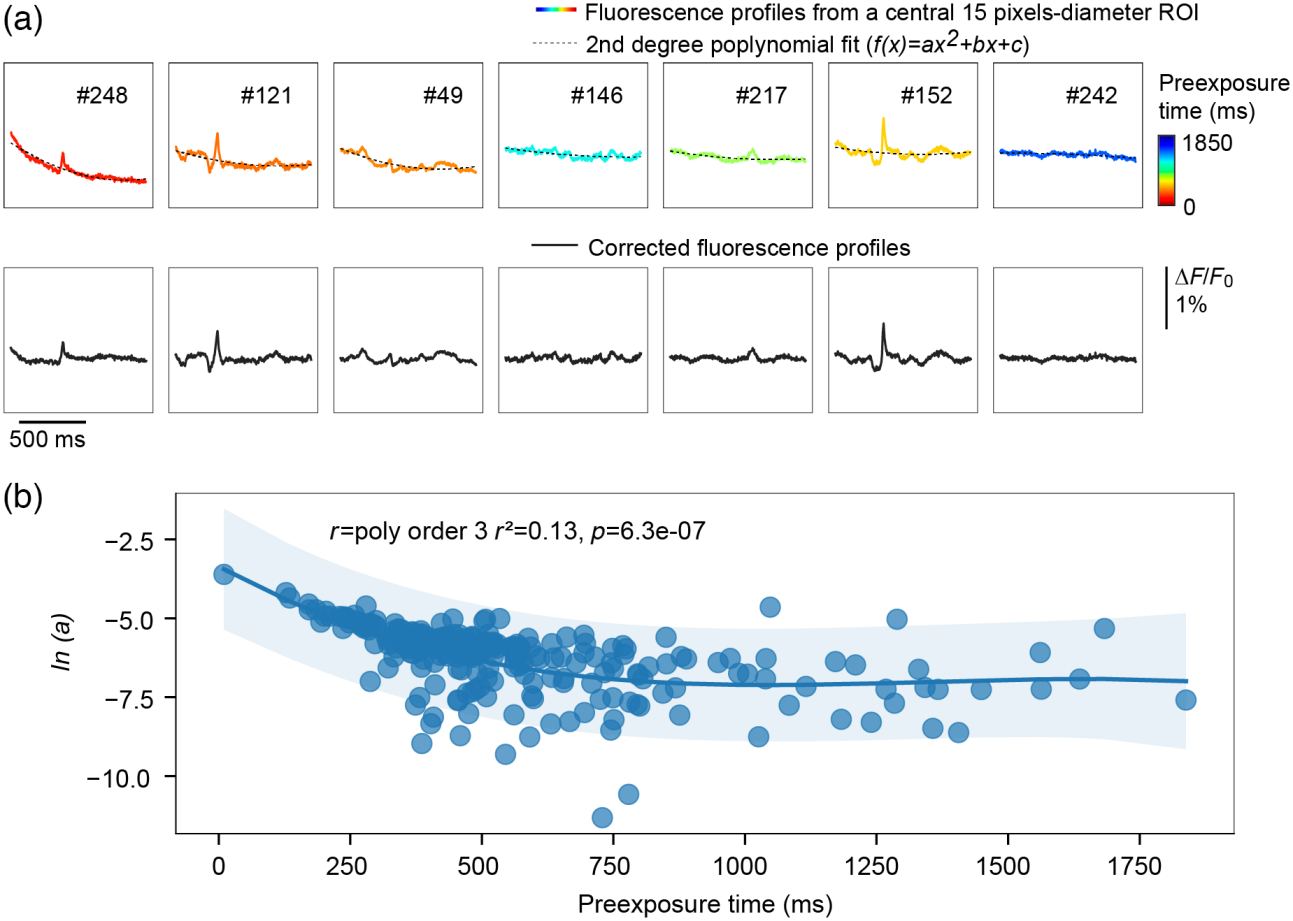
Photobleaching of the VSD fluorescence and its off-line correction. (**a**) Fluorescence profiles computed form a central 15 pixels-diameter region of interest (ROI) from some example trials of the freely moving VSD recording session illustrated in Fig. 5. The trial number is indicated above each trace. After a 2.5-Hz lowpass Butterworth filtering, the fluorescence profiles were fitted with a second degree polynomial function (black dotted lines). Traces are sorted according to the leading term of the polynomial fit (a, higher values to the left), and color-coded depending on the time elapsed between the opening of the shutter and the start of the image sequence acquisition (shorter pre-exposition time toward red colors). Bottom panel: Corrected fluorescence profiles. (**b**) For each trial of the session, the natural log of the leading term of the polynomial fit (a) is plotted against the pre-exposure time revealing an accentuation of the photobleaching curve for shorter pre-exposure times. The curve represents the polynomial regression prediction fitted from the data, and the shaded area represents the confidence interval of that prediction.

Reversible photobleaching of the RH1691 dye follows an exponential shape occurring at the second timescale, it is thus necessary to correct signals for this rapid decay. In this purpose, for experiments focusing on the activity evoked by a given stimulus with anesthetized animals, it is classical to subtract signals acquired in “blank” trials devoid of stimulation, from trials where the stimulus was delivered. However, when working with awake freely moving animals, such a procedure cannot be considered. By working with a substantial pre-exposure time, the acquisition starts as the steeper phase of the bleaching is passed, and as a result, it is easier to get rid of the bleaching artefact following the method described in the Sec. 2.8.4. Our bleaching correction method is illustrated for few example trials in Fig. 6 (same mouse as for Fig. 5). This figure also illustrates how the shape of the decay of fluorescence varies with the pre-exposure time.

### 5.2 Appendix B: Spatiotemporal Dynamics of VSD Fluorescence Signals Recorded in Response to Single Whisker Stimulations Through the Fibroscope

**Fig. 7:**
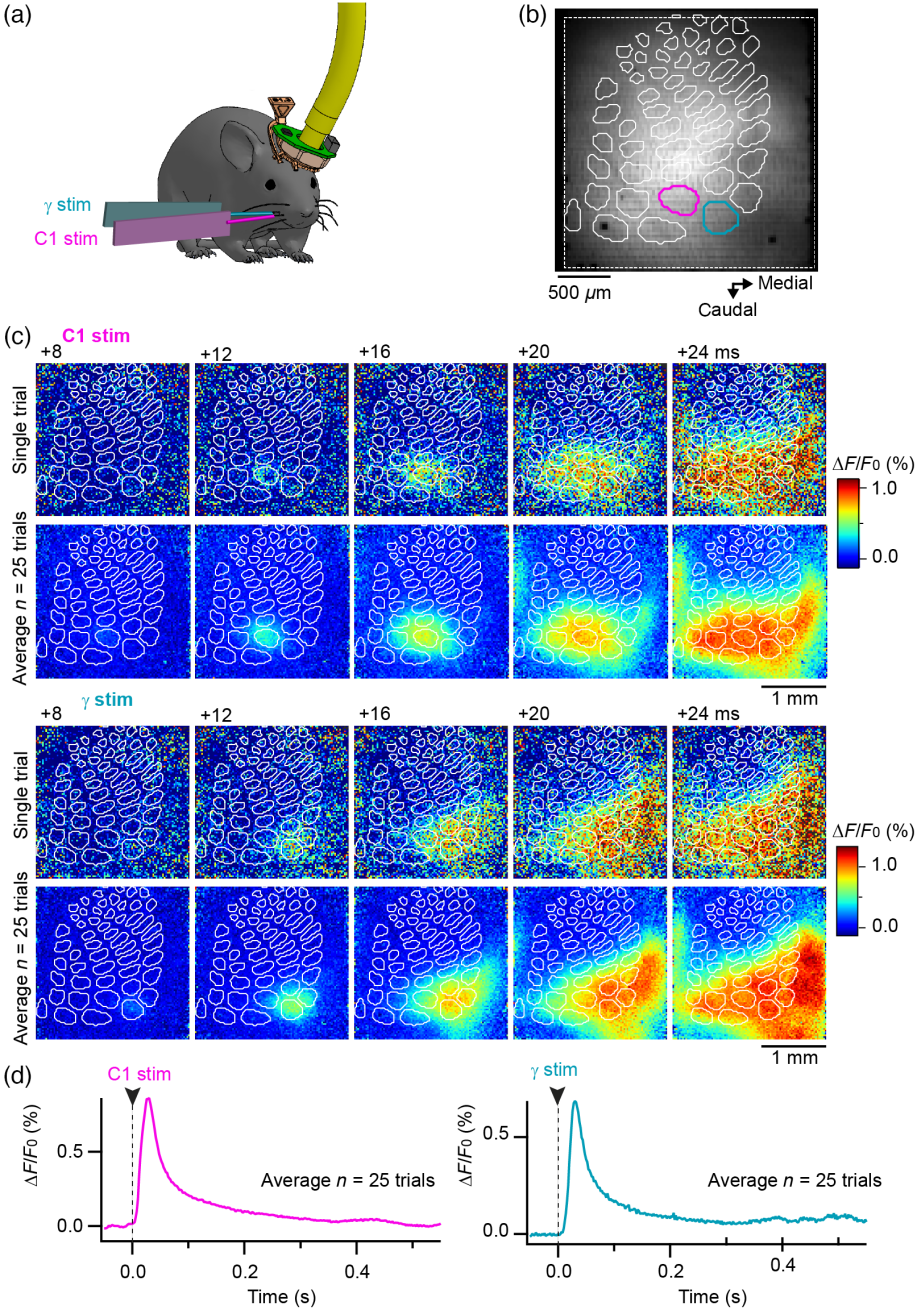
Spatiotemporal dynamics of VSD fluorescence signals recorded in response to single whisker stimulations through the fibroscope. (**a**) At the end of the recording session performed as the animal was engaged in the whisker-guided locomotion task (illustrated in Fig. 5), the mouse was fixed by its implant and anesthetized with isoflurane. Fluorescent signals evoked by a single deflection (2 ms) of the C1 and γ whiskers were then imaged through the fibroscope. (**b**) Image of the resting fluorescence together with the layer 4 barrel map of vS1 reconstructed from a posthoc cytochrome oxidase staining (white contours, the C1 and γ barrels are shown in corresponding colors). (**c**) Snapshots of relative changes in VSD fluorescence captured at different time points after stimulation of the whiskers C1 (top panel) or γ (bottom panel). Representative trials are shown above averages of n=25 trials. (**d**) Corresponding averaged fluorescence profiles quantified from ROIs defined from the C1 and γ barrel delimitations, respectively.

At the end of the freely moving imaging session, mice were anesthetized with isoflurane, and whisker-evoked activity was imaged through the fibroscope kept in place. These signals (illustrated in Fig. 7 for the same mouse as in Fig. 5) were used to assess for the correct realignment of the histologically reconstructed barrel map with the functional VSD images (as described in Ref. 41). They also demonstrate the quality of the signals collected through the fibroscope equipped with our optical rotary joint.

### 5.3 Appendix C: Patterned Noise Correction with an Inverted Fast Fourier Transform

**Fig. 8:**
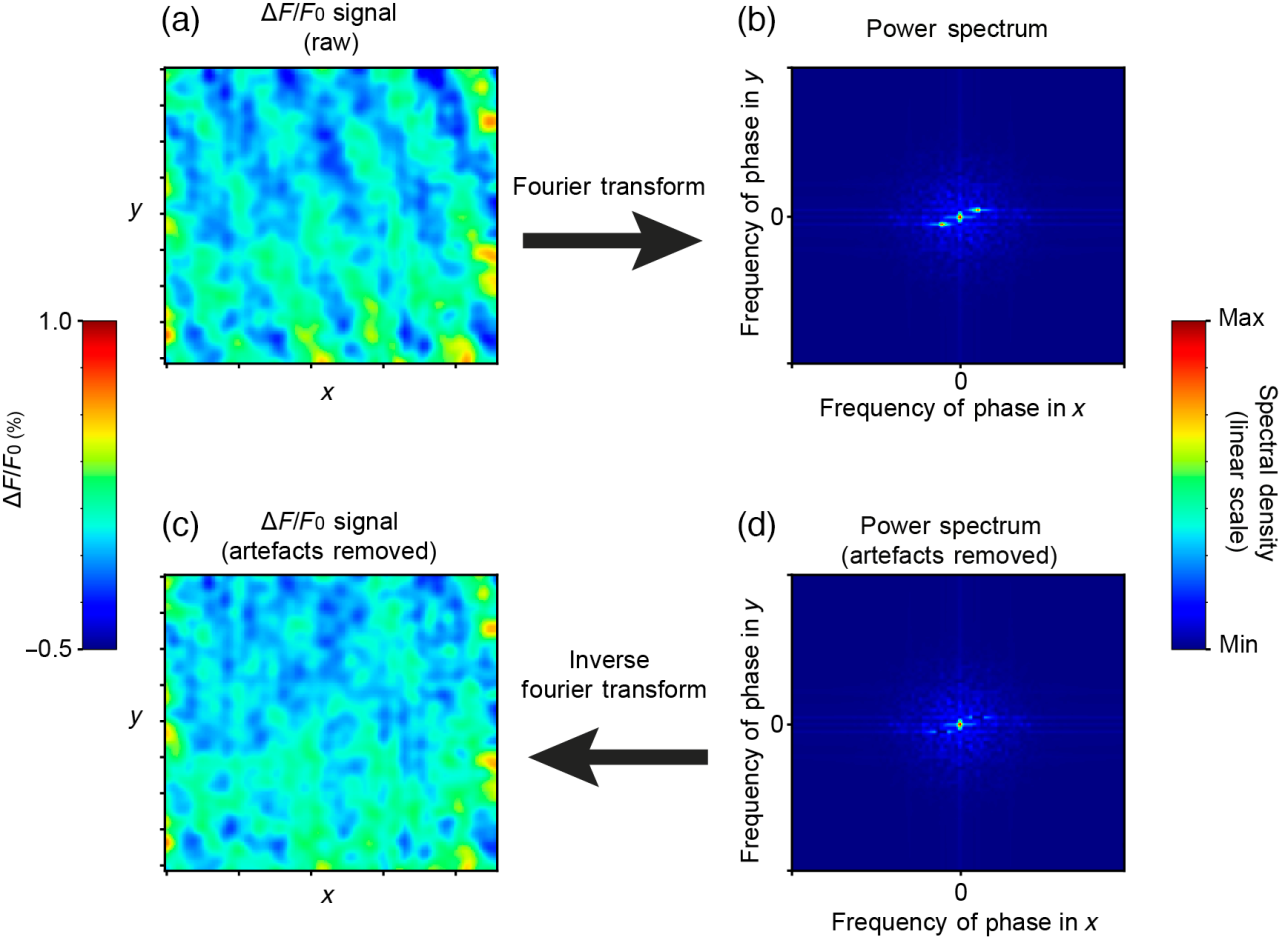
Patterned noise correction with an inverse Fourier transform (iFFT). Step by step procedure used to filter out a visible patterned noise that can be seen in raw ΔF/F0 signal. This artefact can be described as specific spatial sine waves (a) present in virtually all recorded trials in the freely moving configuration and easily identifiable when viewing the Fourier transform of the signal in space (b). To correct this artefact, its phase orientation and spatial frequency are first visually identified on the Fourier transform for multiple frames, and then the problematic phase-frequency orientation is set to 0 for every recorded frame (d). We then perform the iFFT on our “cleaned” frequency spectrum to obtain a “cleaned” ΔF/F0 signal (c). Note that the spatial frequency and phase orientation of this patterned noise remained identical across mice and recording sessions.

To filter out a visible patterned noise that we observed in our raw $\Delta F/F_{0}$ signal, we used a iFFT procedure illustrated in Fig. 8.

### 5.4 Appendix D: Additional Examples of VSD Signals Recorded Through the Fibroscope

**Fig. 9:**
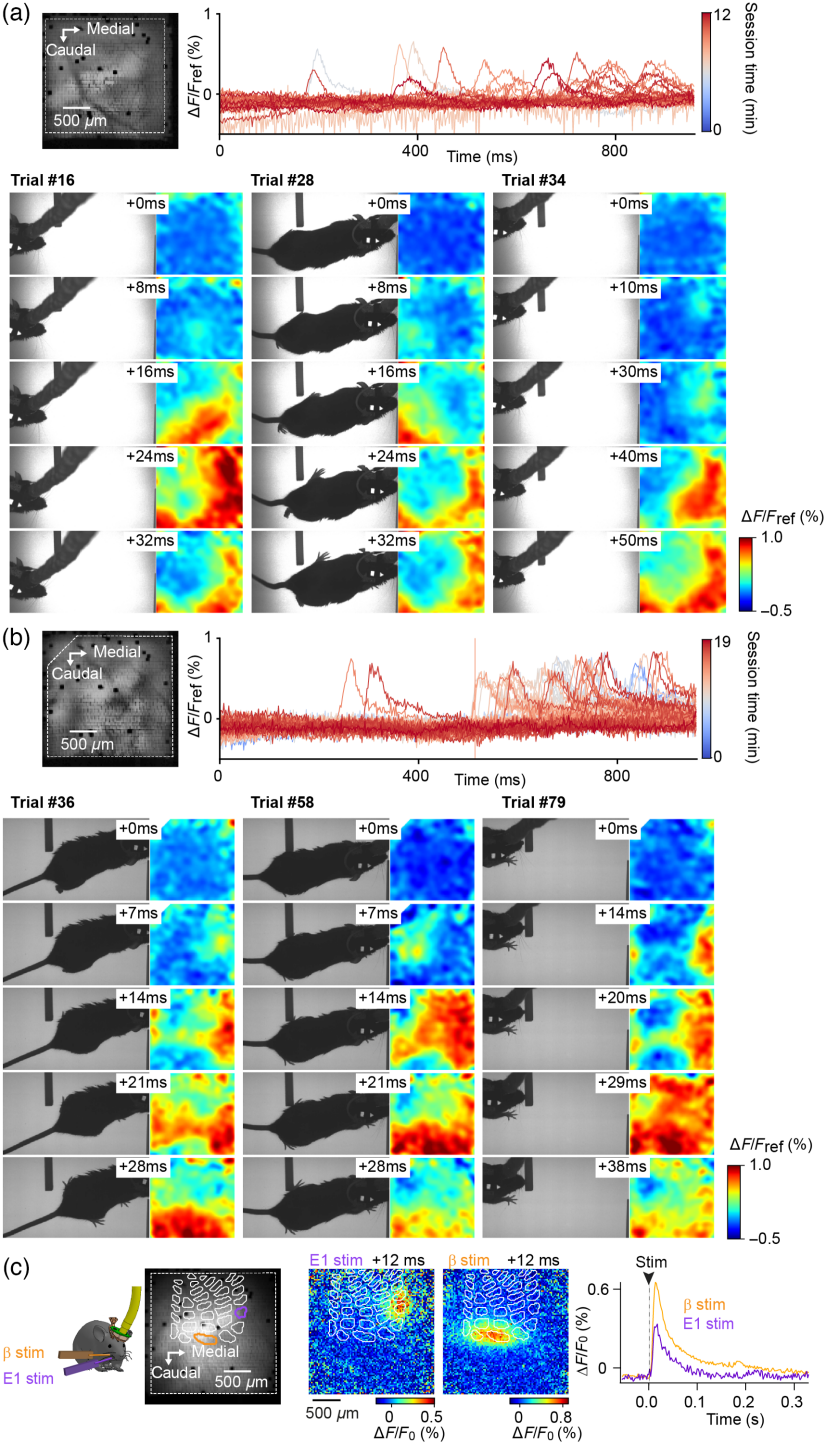
Additional examples of VSD signals recorded through the fibroscope. (**a,b**) Two additional examples of VSD imaging of cortical dynamics during execution of a whisker-guided locomotion task. Top panels: resting VSD fluorescence imaged through the fiber-bundle at OREF (left) and variation of VSD fluorescence quantified from a large ROI (dashed white outline on the left image) for all the trials of the session (from the first trial in dark blue, to the last trial in dark red). Bottom panels: snapshots of the infrared behavior video and corresponding relative variations of fluorescence ($\Delta F/F_{\mathrm{ref}}$, $F_{\mathrm{ref}}$ being an average of three frames centered at time 0 of a given wave of activity), captured synchronously in the straight lane of the track, as the mouse passes by an obstacle. (**c**) VSD signal evoked by a single deflection (2 ms) of the β and E1 whiskers was imaged through the fibroscope, under isoflurane anesthesia, at the end of the recording session performed as the animal was engaged in the whisker-guided locomotion task [same mouse as in panel (b)]. Resting fluorescence together with the reconstructed barrel map is shown on the left. Snapshots taken 12 ms following whisker stimulation are shown in the middle and corresponding profiles of fluorescence are shown on the right (averages of n=15 trials for each condition).

Cortical dynamics recorded during execution of the whisker-guided locomotion task are illustrated in Fig. 5 for one animal and data from two other experiments are shown in Fig. 9.

### 5.5 Appendix E: Stability of the Recordings During Execution of Whisker-Guided Locomotion Task

**Fig. 10:**
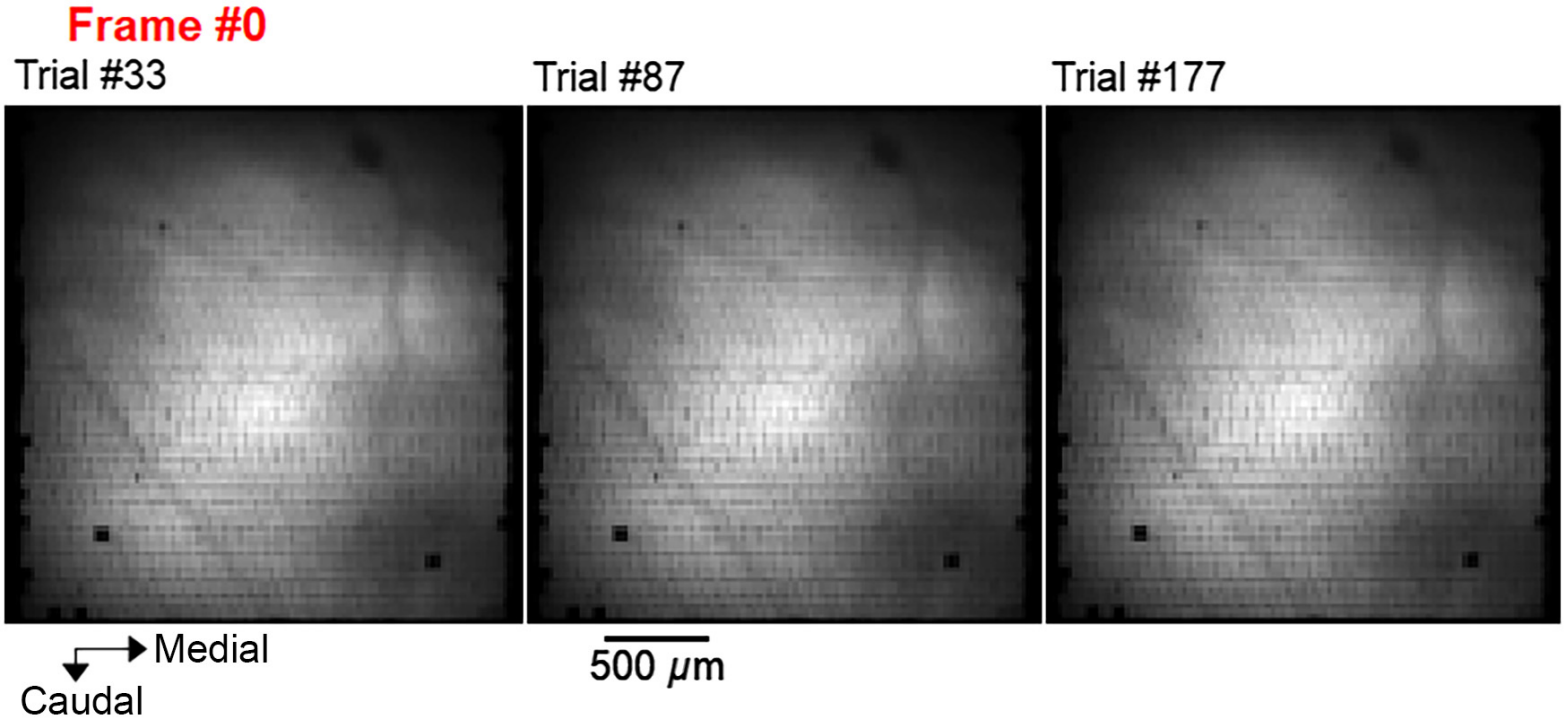
Absolute fluorescence signals recorded during the three trials from which the relative changes of fluorescence (functional signals) are illustrated in Fig. 5 (Video 1, MOV, 8.67 MB [URL: https://doi.org/10.1117/1.NPh.10.1.015009.s1]).

Visualization of absolute VSD fluorescence signals acquired through the fibroscope during individual image sequences (trials) demonstrates the stability of the images despite the animal running in the behavioral arena (Fig. 10).

## Code, Data, and Materials Availability

Information regarding the operation, building instruction, and code of the device is accessible on an open repository, https://github.com/ShulzLab/MORJ. The data that support the findings of the study are available upon request.
